# The Relationship between Cerebral Glucose Metabolism and Age: Report of a Large Brain PET Data Set

**DOI:** 10.1371/journal.pone.0051517

**Published:** 2012-12-20

**Authors:** Xiaoyan Shen, Huafeng Liu, Zhenghui Hu, Hongjie Hu, Pengcheng Shi

**Affiliations:** 1 State Key Laboratory of Modern Optical Instrumentation, Department of Optical Engineering, Zhejiang University, Hangzhou, China; 2 B. Thomas Golisano College of Computing and Information Sciences, Rochester Institute of Technology, Rochester, New York, United States of America; 3 Department of Radiology, Sir Run Run Shaw Hospital, College of Medicine, Zhejiang University, Hangzhou, China; Institution of Automation, CAS, China

## Abstract

Cerebral glucose metabolism is a reliable index of neural activity and may provide evidence for brain function in healthy adults. We studied the correlation between cerebral glucose metabolism and age under the resting-state in both sexes with position emission tomography. Statistical test of age effect on cerebral glucose metabolism was performed using the statistical parametric mapping software with a voxel-by-voxel approach (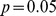 family wise error corrected, 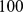-voxel threshold). The subjects consisted of 108 females (mean 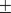 S.D. = 45
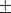
10 years) and 126 males (mean 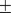 S.D. = 49
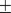
11 years). We showed here that brain activity in the frontal and temporal lobes in both sexes decreased significantly with normal aging. The glucose metabolism in the caudate bilaterally showed a negative correlation with age in males, but not in females. Few regions in males were shown with an increased glucose metabolism with age. Although the mechanisms of brain aging are still unknown, a map of brain areas susceptible to age was described in this report.

## Introduction

It is now well established that normal aging is associated with a progressive decline in cognitive performance, including perception, attention, language and memory [1]–[4]. Although the causes of age-related cognitive decline remain elusive, many studies point out that the normal aging is accomplished by a decline of synaptic activity, which may impact the cognitive functions [5]–[9]. Parkin et al. [6] and Miller et al. [7] found that age-related memory deficits were associated with a decreased neuronal activity in frontal and temporal lobes, and the influences of hippocampal synaptic activity on memory impairment had also been observed by Geinisman et al. [5]. Moreover, Pardo et al. [8] reported a positive correlation between the neuron activity in the prefrontal lobe and semantic fluency. Therefore, understanding age-related brain activity changes is essential for assessing the elderly presenting with cognitive complaints and preventing potential cognitive impairment with aging.

In the resting state, the level of cerebral glucose metabolism is considered as a reliable index of neural activity [10]. Synapses are the key sites for information transfer between neurons in the brain. Phelps et al. [11] studied the cerebral metabolic rate of glucose in resting state and found that up to 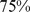 of the glucose consumption in the brain was used to maintain a baseline synaptic activity. Therefore, measuring the resting cerebral glucose metabolic with positron emission tomography (PET) is available for detecting age-related brain activity changes. In the past few years, considerable efforts have been done in studying the age-related brain activity changes by measuring the resting cerebral metabolic rate of glucose with [^18^F] fluoro-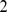-deoxyglucose (FDG)-PET and an age-related glucose metabolism decline prominently appears in the frontal lobe [8], [10], [12]–[16].

However, previous reports about the relationship between the regional cerebral glucose metabolism and age are discrepant in a number of brain areas, sometimes conflicting. For example, one study indicated a decreased glucose metabolism with age in the thalamus [8], while Willis et al. reported an increased glucose metabolism in the same region [16]. The divergence in results may be due to the different methodologies, screen criteria and range of subject ages, especially the sample size which is one of the key issues to obtain consistent, statistical results. On the other hand, most early studies on age-related glucose metabolism use region of interest (ROI) analysis [15], . In recent years, voxel-based quantitative analysis methods such as statistical parametric mapping (SPM) have been widely used [8], [10], [16]. SPM offers a statistical mapping of whole brain by an automated and voxel-based analysis, which helps to detect the areas missed in region of interest (ROI) analysis and avoid subjectivity variation. Here, we used resting state FDG-PET data from a large sample of health adults (in total 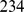 subjects) across a wide range of age, analyzed by SPM to identify the correlation of the regional cerebral glucose metabolism changed with normal aging. As various studies have reported the sex differences in brain function [4], [23], [25], it is reasonable to analyze the age effects on regional cerebral glucose metabolism for females and males separately. We expect to find a consistency effect of aging on the regional brain activities.

## Materials and Methods

### Subjects

We studied 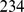 consecutive subjects from a clinical database, and all subjects were gave written informed consent for their information to be used for the future research. The study was reviewed and approved by the ethics committee of Zhejiang University and the experiments were conducted according to the Declaration of Helsinki. The subjects consisted of 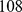 females aged from 26 to 71 years (mean 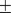 S.D. 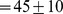 years) and 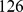 males aged from 28 to 77 years (mean 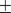 S.D. 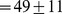 years). All subjects had a normal physical examination before imaging. The subjects were selected according to the following criteria: no significant acute or chronic disease was found at the time of the study. Subjects reported no history of brain injury, neurological illness or clinical evidence of significant cognitive decline beyond the expected for normal aging.

### PET imaging

All PET investigations were performed at the Medical PET Center of Zhejiang University. PET images were acquired with Hamamatsu SHR 22000 whole-body PET scanner in two-dimensional mode. The scanner has a 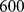 mm patient aperture and an axial field-of-view of 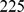 mm, which can cover the whole human head. The spatial resolution of the scanner is 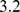 mm full width at half maximum (FWHM) in axial plane and 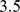 mm FWHM in sagittal or coronal plane. A 10-min transmission scan was performed before the emission scan using a ^68^Ge source for attenuation correction. All subjects rested in a quiet, dark room with eyes closed and ears open for 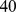 min after 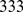
–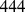 MBq (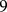
–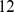 mCi) FDG was injected intravenously. Then emission scans were acquired under the resting state (lying quietly with eyes closed) for 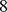 minutes. PET images were reconstructed with a ramp filter to the Nyquist frequency, using the maximum-likelihood expectation maximization (MLEM) algorithm.

### Data analysis

The tissue concentration of FDG can be calculated from the pixel intensity values of the PET images as described by Kumar et al. [26] The regional cerebral metabolic rates for glucose were derived form the relationship between the tissue concentration and the integrated plasma levels of FDG by using a modified Sokoloff equation [27]. Therefor, the regional cerebral metabolic rates for glucose can be represented by pixel intensity values of the PET images, which can be quantitatively used for statistical analysis. PET images were analyzed using matlab 6.5 (MathWorks Inc., Notich, MA, USA) and Statistical Parametric Mapping (SPM5, Wellcome Department of Cognitive Neurology, London, UK) software. Prior to statistical analysis, raw PET data were converted into Analyze format using ImageJ (Wayne Rasband, National Institute of Mental Health, USA) and MRIcro software (www.mricro.com). All PET images were spatially normalized into the Montreal Neurological Institute (MNI, McGill University, Montreal, Canada) standard template using SPM5. After normalization, spatial smoothing was performed by convolution, using an isotropic Gaussian kernel with 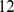 mm FWHM to increase the signal-to-noise ratio. All subjects were separated into two groups (the female group and the male group) and analyzed respectively. Statistical test of age effect on cerebral glucose metabolism was performed through voxel-based analysis using a general liner model (GLM). In the GLM analysis, age was a covariate to study the relationship between glucose uptake and normal aging. T-test was used to examine the regression coefficient. Global nuisance effects were eliminated by including the global covariate in the general linear model. In SPM maps, we searched the brain areas with a significant correlation using a statistical threshold of 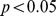, family wise error (FWE)-corrected for the problem of multiple comparisons, with an extent threshold of 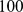 voxels. For the whole brain of 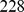 resels, the uncorrected 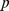 values were 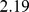
e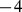. The significant areas were overlaid on a T1-weighted MRI image slice by slice. The MNI coordinates were converted to the Talairach coordinates, and the Talairach Client was used for localization. In order to measure the effect of age on regional cerebral glucose metabolism quantitatively, correlation analysis were obtained by calculating the Pearson correlation coefficient (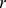) for each significant cluster. Scatter plotted using local maximum activity of each cluster versus age.

## Results

### Effects of aging on glucose metabolism in the female group

**Figure 1:**
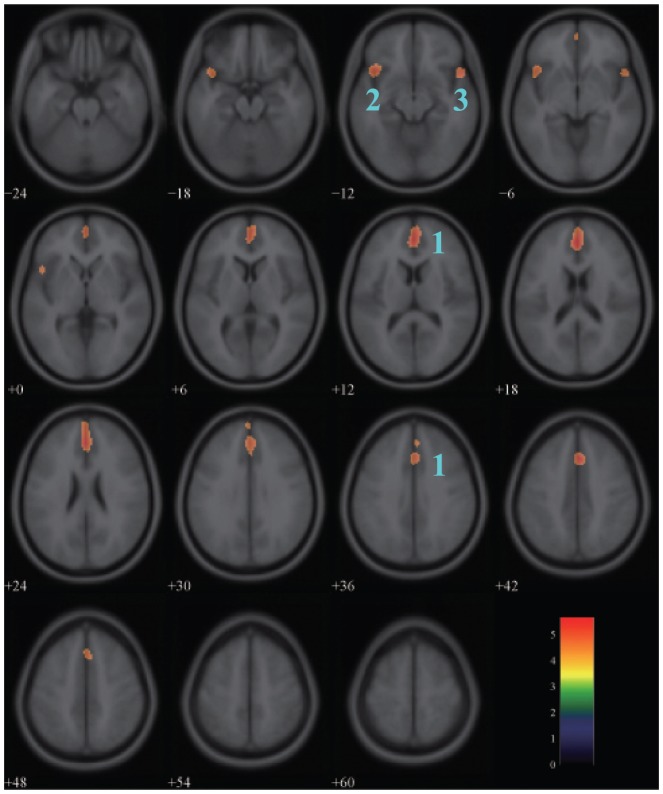
Areas of negative correlation between glucose metabolism and age in the female group. The significant areas overlaid on a T1-weighted MRI image are displayed with a statistical threshold of 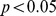 FWE-corrected and an extend threshold of 100 voxels. The number of slices correspond to the z values in Talairach coordinate system which defined form inferior to superior. Clusters 1–3 represent the left medial frontal gyrus
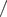
right cingulate gyrus, the left inferior frontal gyrus and the right superior temporal gyrus respectively. Color scale denotes 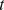 value.

The result of one-sample 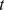-test of correlation between glucose metabolism and age in the female group was shown in Figure 1. The map illustrated the regions of negative correlation with a statistical threshold of 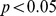 FWE-corrected and an extend threshold of 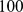 voxels. Three clusters in the frontal lobe and the temporal lobe showed the significant negative correlations: the left medial frontal gyrus (BA 9, 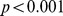 FWE-corrected, 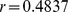), the left inferior frontal gyrus (BA 47, 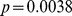 FWE-corrected, 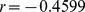) and the right superior temporal gyrus (BA 38, 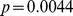 FWE-corrected, 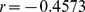). More detailed information was listed in Table 1.

**Table 1:** Clusters of glucose metabolism decrease with normal aging in the female group. BA, Brodmann area; (x,y,z), local maximum activity of cluster in Talairach coordinate system; 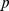, significance level; 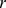, Pearson correlation; Voxels, number of voxels within cluster; L, left; R, right.

Cluster	Region name	BA	Talairach coordinates 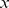 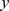 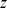 (mm mm mm)			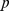 FWE-corr	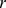	Voxels
1	Medial frontal gyrus (L)	9	0	43	13	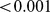	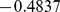	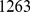
	Cingulate gyrus (R)	32	4	23	36	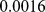	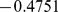	
2	Inferior frontal gyrus (L)	47	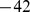	17	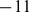	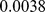	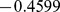	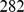
3	Superior temporal gyrus (R)	38	51	13	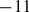	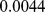	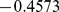	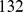

**Figure 2:**
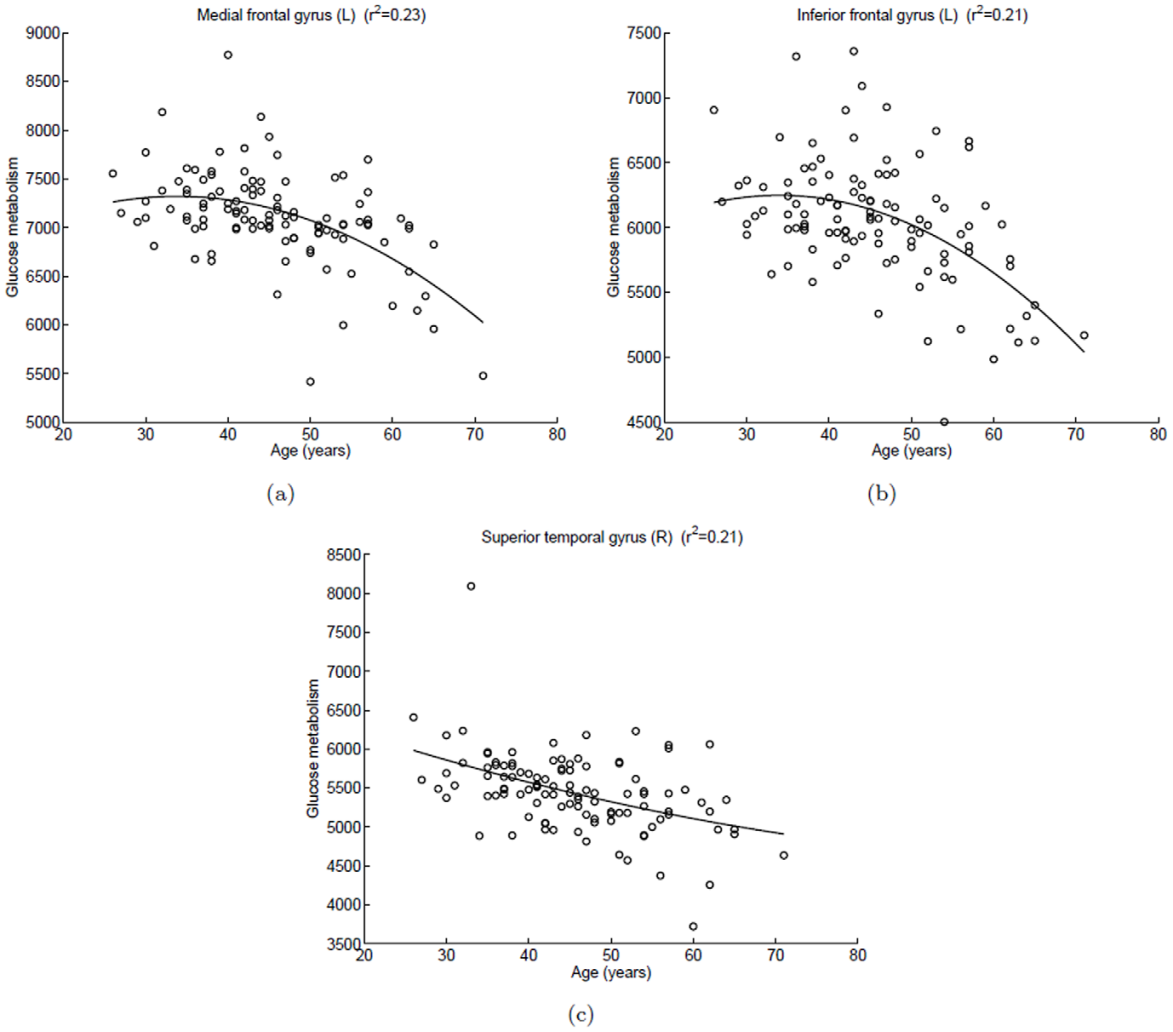
Scatterplots of glucose metabolism in local maximum of each cluster versus age in the female group. A quadratic polynomial fitting is used to express the relationship between glucose uptake and age. (a) Left medial frontal gyrus, 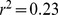; (b) Left inferior frontal gyrus, 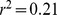; (c) Right superior gyrus, 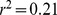.

Figure 2 showed the scatterplots of glucose metabolism in local maximum of each cluster versus age, and a quadratic polynomial fitting was used to express a trend of decreased glucose uptake with aging. For the left medial frontal gyrus and the left inferior frontal gyrus, the curves were nearly horizontal before 40 years of age, and after that the curve appears an accelerated declining trend. For the right superior temporal gyrus, the curve remained an almost constant non-zero curvature and slow downward trend.

In the female group, regions of positive correlation between glucose metabolism and age were not found.

### Effects of aging on glucose metabolism in the male group

**Figure 3:**
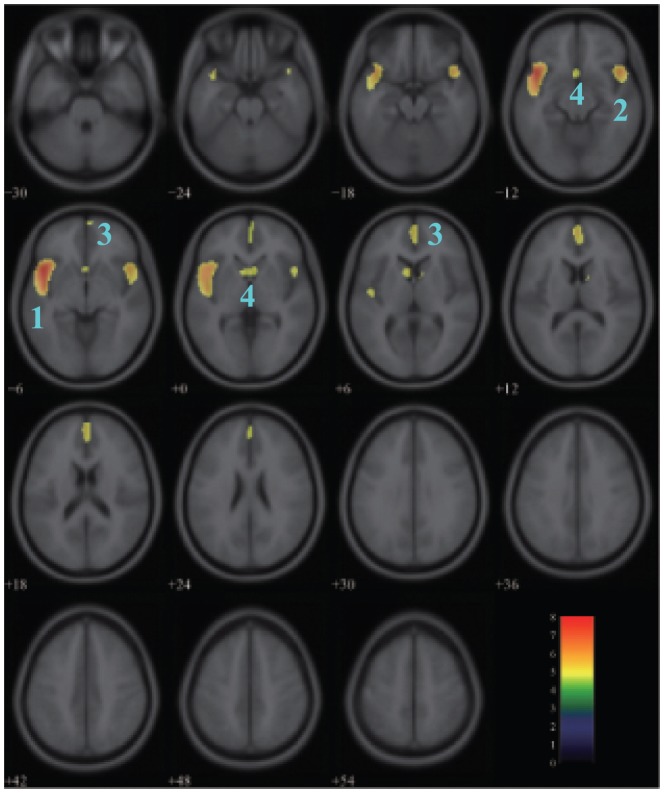
Areas of negative correlation between glucose metabolism and age in the male group. The significant areas overlaid on a T1-weighted MRI image are displayed with a statistical threshold of 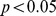 FWE-corrected and an extend threshold of 100 voxels. The number of slices correspond to the z values in Talairach coordinate system which defined form inferior to superior. Clusters 1–4 represent the left superior temporal gyrus, the right superior temporal gyrus, the medial frontal gyrus, and the caudate
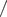
left subcallosal gyrus respectively. Color scale denotes 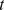 value.

Figure 3 showed the areas of negative correlation between glucose metabolism and age in the male group with the same threshold set as above. Areas of relative decreased glucose uptake with age emerged bilaterally in the superior temporal gyrus (BA 38, 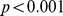 FWE-corrected, 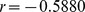 for the left and 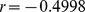 for the right), the medial frontal gyrus (BA 10, 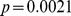 FWE-corrected, 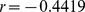 for the left; 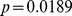 FWE-corrected, 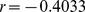 for the right) and the caudate (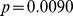 FWE-corrected, 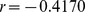 for the left; 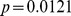 FWE-corrected, 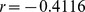 for the right) and in the left subcallosal gyrus (BA 25, 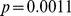 FWE-corrected, 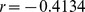). Table 2 listed the detailed information for each cluster.

**Table 2:** Regions of glucose metabolism decrease with normal aging in the male group. BA, Brodmann area; (x,y,z), local maximum activity of cluster in Talairach coordinate system; p, significance level; r, Pearson correlation; Voxels, number of voxels within cluster; L, left; R, right.

Cluster	Region name	BA	Talairach coordinates 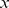 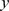 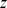 (mm mm mm)			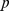 FWE-corr	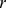	Voxels
1	Superior temporal gyrus (L)	38	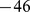	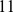	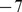	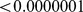	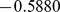	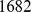
2	Superior temporal gyrus (R)	38	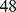	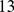	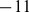	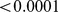	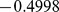	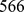
3	Medial frontal gyrus (L)	10	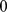	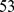	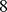	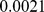	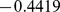	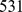
	Medial frontal gyrus (R)	10	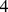	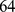	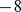	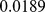	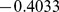	
4	Caudate (L)		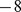	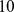	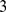	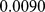	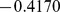	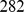
	Subcallosal gyrus (L)	25	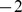	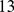	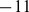	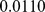	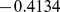	
	Caudate (R)		6	12	1	0.0121	−0.4116	

**Figure 4:**
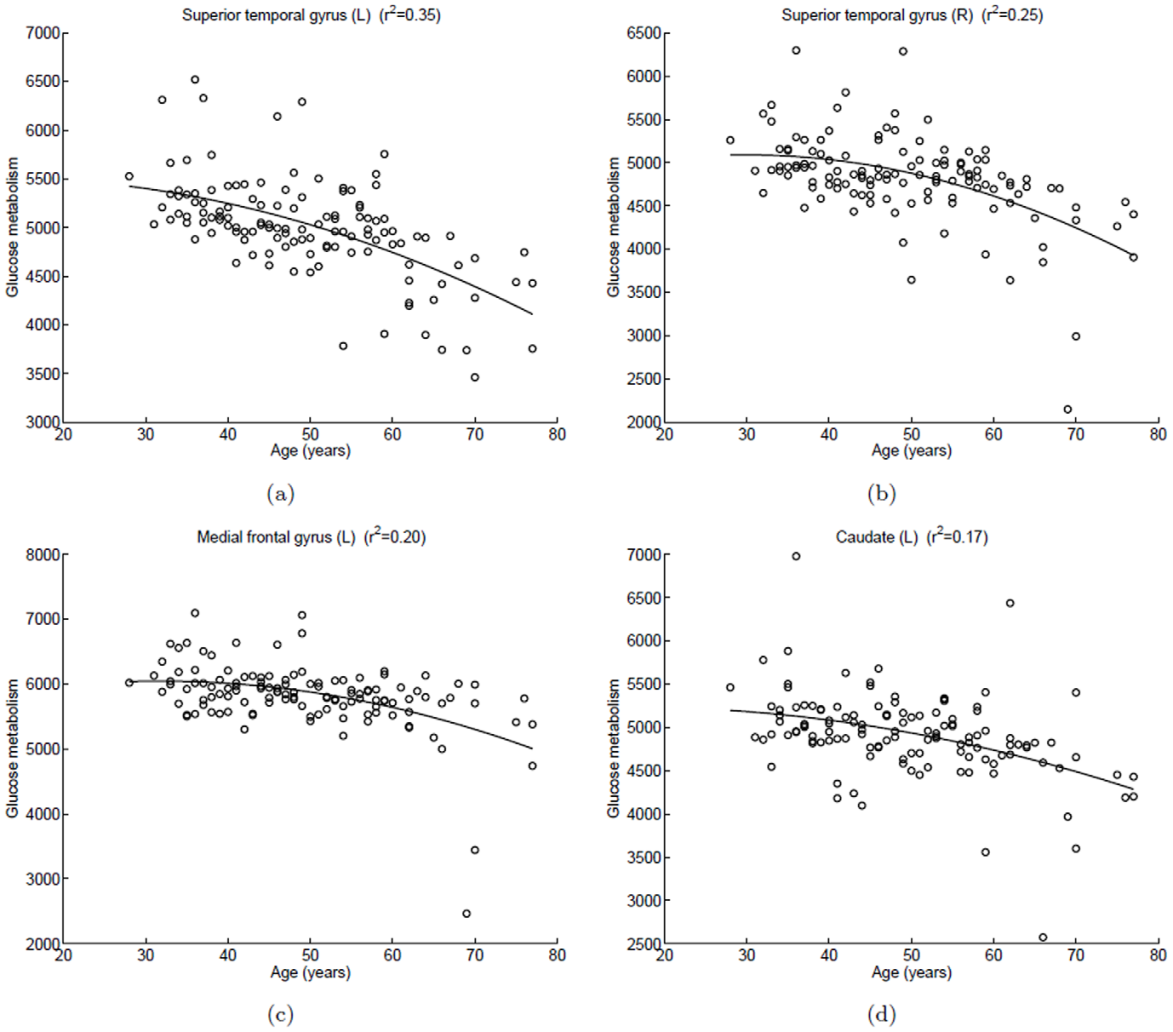
Scatterplots of glucose metabolism in local maximum of each cluster versus age in the male group (negative correlation). A quadratic polynomial fitting is used to express the relationship between glucose uptake and age. (a) Left superior temporal gyrus, 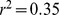; (b) Right superior temporal gyrus, 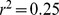; (c) Left medial frontal gyrus, 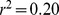; (d) Left caudate, 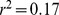.

In Figure 4, scatterplots of glucose metabolism in local maximum of each cluster versus age were fitted with quadratic polynomial method. As shown in Figure 4 (a) and (b), the decline with aging in the left superior temporal gyrus was steeper than in the right superior temporal gyrus. For each of the four clusters, the curvature of curve increased with aging.

**Figure 5:**
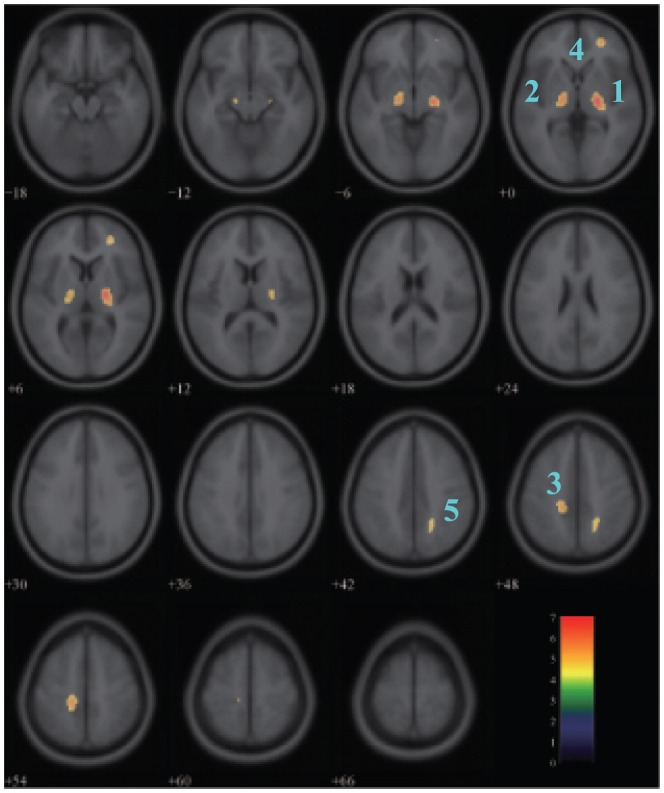
Areas of positive correlation between glucose metabolism and age in the male group. The significant areas overlaid on a T1-weighted MRI image are displayed with a statistical threshold of 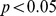 FWE-corrected and an extend threshold of 100 voxels. The number of slices correspond to the z values in Talairach coordinate system which defined form inferior to superior. Clusters 1–5 represent the right lentiform nucleus, the left thalamus, the left paracentral lobule, the right middle frontal gyrus and the right precuneus respectively. Color scale denotes 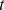 value.

**Figure 6:**
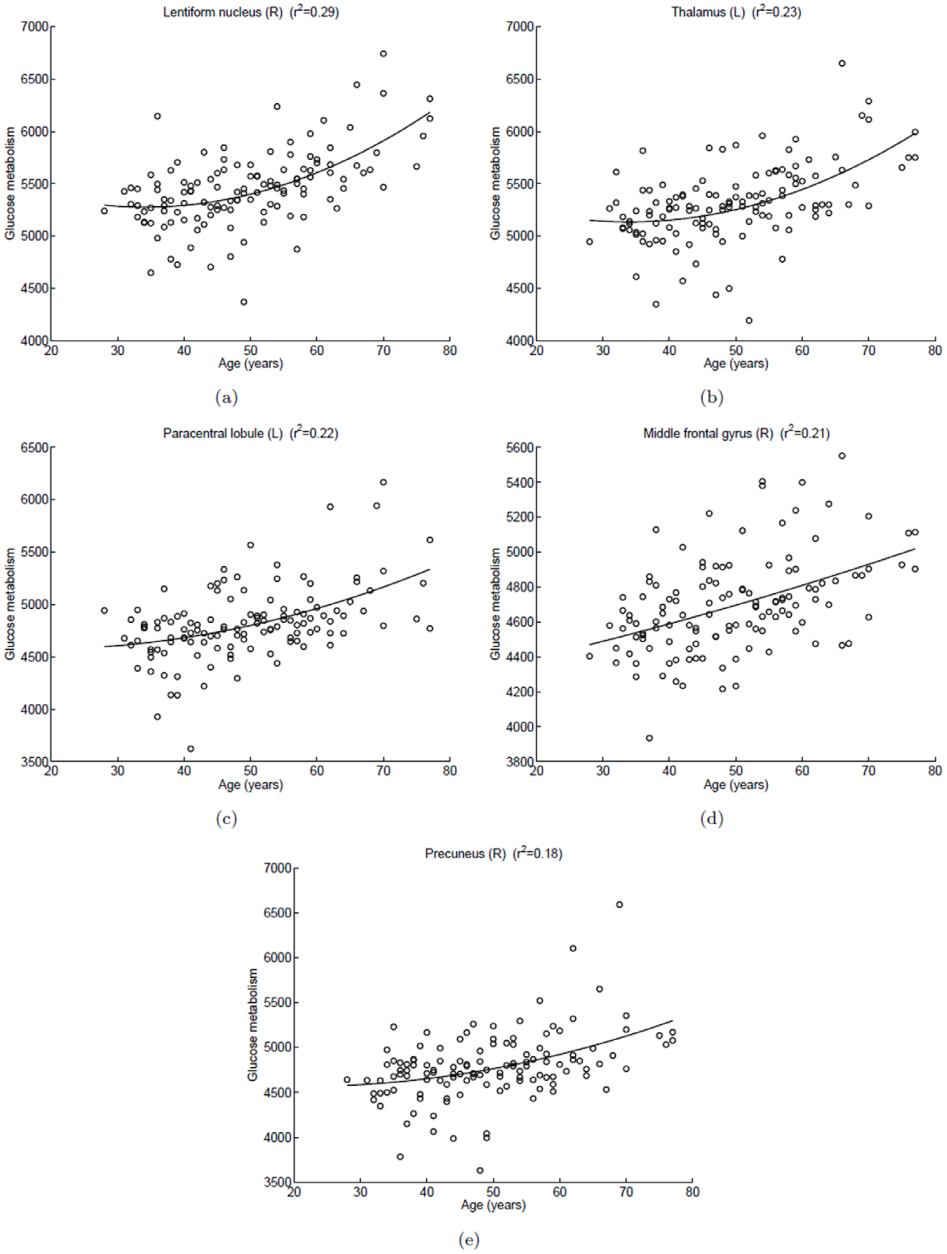
Scatterplots of glucose metabolism in local maximum of each cluster versus age in the male group (positive correlation). A quadratic polynomial fitting is used to express the relationship between glucose uptake and age. (a) Right lentiform nucleus, 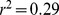; (b) Left thalamus, 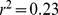; (c) Left paracentral lobule, 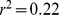; (d) Right middle frontal gyrus, 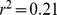; (e) Right precuneus, 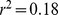.

In the male group, significant age-related increasing in glucose metabolism was evident in the lentiform nucleus in the right sub-lobar (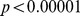 FWE-corrected, 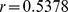) (Figure 5, Table 3). The positive correlations were also found in the left thalamus, the left paracentral lobule (BA 5), the right middle frontal gyrus (BA 10) and the right precuneus (BA 7). Figure 6 showed scatterplots of glucose metabolism versus age in the brain area with positive correlation.

**Table:** **Table 3.** Regions of glucose metabolism increase with normal aging in the male group. BA, Brodmann area; (x,y,z), local maximum activity of cluster in Talairach coordinate system; p, significance level; r, Pearson correlation; Voxels, number of voxels within cluster; L, left; R, right.

Cluster	Region name	BA	Talairach coordinates x y z (mm mm mm)			p FWE-corr	r	Voxels
1	Lentiform nucleus (R)		22	−15	3	< 0.00001	0.5378	460
2	Thalamus (L)		−16	−14	−1	< 0.001	0.4802	289
3	Paracentral lobule (L)	5	−16	−29	47	< 0.001	0.4738	222
4	Middle frontal gyrus (R)	10	28	47	−2	< 0.001	0.4594	131
5	Precuneus (R)	7	20	−46	43	0.0055	0.4257	110

## Discussion

Age related gyral narrowing and sulcal widening can cause artifacts in glucose metabolism. Some studies found that a decline glucose uptake with normal aging becomes insignificant after taking the partial volume effects into account [28], . However, the structure atrophy is not able to fully explain the age related declines in glucose metabolism. First, studies that investigated the cortical changes across the life span showed a prominent atrophy in the prefrontal cortex, while the cortical thinning in the temporal cortex was noted to a less extent [30], [31]. Furthermore, in the frontal cortex, the age effects on structural atrophy [31] showed a different pattern from the age effects on hypometabolism in our study (Figure 2 and Figure 4). With increasing age, gray matter intensity declines quickly before 40 years of age and then remains relatively stable [31], while in our study the glucose metabolism keep stable before age 40 and then acceleration decrease. This suggests that the results we reported at least partially reflect the declines in glucose metabolism.

In our analysis of the resting-state PET images, the glucose metabolism in the frontal lobe declines with normal aging in both the female and the male groups. It is consistent with the results of prior studies which have also used a voxel-based analysis [8], [14], [16]. The frontal lobe plays a key role in reasoning, planning, language, attention, emotions and movement [8], [32], [33]. The decline of glucose metabolism with normal aging in the frontal lobe may explain why the mental efficiency reduces in the elderly.

The decline of glucose metabolism in the superior temporal gyrus with aging is significant in both sexes. Willis et al. [16] reported a similar correlation in cerebral glucose uptake with aging. Comparing the Figure 2 (c) with Figure 4 (c) and (d), we find the decline trend of glucose metabolism in the male group is faster than in the female group in the temporal lobe. In prior MRI studies, Murphy et al. [23] and Gur et al [34] reported that the age-related brain atrophy in men is greater than in women, which may explain the result obtained in our statistical analysis. Another interpretation for this sex-related variance is that men tend to be more prone to age-related cognitive decline than women. However, more experiments are needed to further clarify this conclusion.

Another observed phenomenon from this study is the asymmetry of the metabolism decline in frontal and temporal lobes, which is predominant in the male group. As described in Figure 1 and Figure 3, the metabolism decline with normal aging in the left cerebrum shows greater significance and larger range compared with the right cerebrum. This result may provide additional evidences to support the model of HAROLD (hemispheric asymmetry reduction in older adults) [35], [36]. HAROLD model states that younger adults show a prominent lateralization of cerebral function in the frontal lobe, but the activity during cognitive performance trends to be more bilateral in older adults, which also happens in the temporal and parietal lobes [37]. According to the research by Reuter-Lorenz et al [38], the elders who displayed a bilateral pattern of activity had a better performance in the verbal working memory task.

A negative correlation between glucose metabolism and age in the caudate is observed in the male group with a less significance relatively. It has been reported by Kawachi et al. [39] in females, however, not in males. The caudate nucleus is a small structure that is located in the subcortical region of the brain. A moderate age-related atrophy of the caudate was studied by [25] using MRI images. The decrease of glucose metabolism with age in the caudate will be resulted by partial volume effects of PET due to the limited spatial resolution.

In the male group, the regions of the lentiform nucleus, thalamus, paracentral lobule, middle frontal gyrus and precuneus show the positive correlation between glucose uptake and age from our studies. The phenomenon can be explained for an increase in activity in these regions to compensate for the other regions of neuron dysfunction or loss. Scarmeas et al. [40] suggested that the age-related increase of metabolism in some regions may be interpreted to compensate for the regions of metabolism decline to cope with the decrease of brain function in a memory condition. The functional preserve is the ability as well as in the resting state. The other interpretation for this aging effect is the different patterns of task performance between the youth and the elderly [32]. As described in the results section, we didn't found regions with positive correlation in the female group. In this study, a statistical threshold of 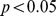 FWE-corrected and an extend threshold of 100 voxels were used for image display and region estimate. When we relax any of these conditions of use, regions with positive correlation appears in the female group. This indicates that both sexes have similar patterns of age-related glucose metabolism but different significance level.

Several limitations of this study should be illustrated. First, as described above, the partial volume effect ignored in this study is a confounding effect in analysis of age-related changes in the metabolism. Another issue is that estrogen use may influence cerebral glucose metabolism in adults [41]. Rasgon et al. found that estrogen use may protect cerebral metabolic from decline in postmenopausal women. We do not exclude the women with estrogen use in this study, which should be considered in the analysis of our results in the female. Further analysis of handedness effects on brain symmetry of glucose metabolism decline are needed to determine whether this is a factor. In addition, since all of the data derived from clinical database, we were unable to take cognitive performance test such as MMSE for each subject at this stage. It will be considered in the future work.
